# Editorial: Dendritic cell-primed T cells in anti-tumor immune responses and relevant vaccine strategies

**DOI:** 10.3389/fimmu.2022.1023967

**Published:** 2022-09-06

**Authors:** Jianmei W. Leavenworth, Xindong Liu, Yunfeng Ma, Yibei Zhu, Chunjian Qi

**Affiliations:** ^1^ Department of Neurosurgery, Heersink School of Medicine, University of Alabama at Birmingham, Birmingham, AL, United States; ^2^ The O’Neal Comprehensive Cancer Center, Heersink School of Medicine, University of Alabama at Birmingham, Birmingham, AL, United States; ^3^ Department of Microbiology, Heersink School of Medicine, Heersink School of Medicine, University of Alabama at Birmingham, Birmingham, AL, United States; ^4^ Institute of Pathology and Southwest Cancer Center, Southwest Hospital, Third Military Medical University (Army Medical University), Chongqing, China; ^5^ School of Basic Medical Sciences, Xi’an Jiaotong University Health Science Center, Xi’an, China; ^6^ Department of Immunology, School of Biology & Basic Medical Sciences, Suzhou Medical College of Soochow University, Suzhou, China; ^7^ Medical Research Center, The Affiliated Changzhou Second People’s Hospital of Nanjing Medical University, Changzhou, China

**Keywords:** dendritic cells, antigen presenting cells, T cells, vaccines, tumor immunity, cancer immunotherapy

Tumor-induced immunosuppression, including T-cell exhaustion, remains one of major hurdles for cancer immunotherapy. Reviving exhausted T-cells to attack tumor is one of major goals of many current immunotherapeutic strategies (1). Priming and sustaining robust T-cell responses require antigen presenting cells (APCs), particularly dendritic cells (DCs). Thus, APC- or DC-based vaccines and related therapeutic strategies against cancer have received much attention (2, 3). Better understanding APC/DC-mediated regulation of tumor immunity would help improve the therapeutic efficacy of the current and future strategies. Our Research Topic has attracted 61 authors who have collectively presented research findings to facilitate our understanding of APC/DC biology, and have provided perspectives on how to exploit these knowledge to develop better APC/DC-based cancer therapies.

DCs are actually heterogenous comprising different subsets with distinct capacity to present or cross-present antigens to T-cells and to regulate immune responses (4, 5). For example, CD8α^+^ DCs (also termed as cDC1) expressing the transcription factor Batf3 are essential for anti-tumor immunity by cross-presenting antigens to cytotoxic CD8^+^ T-cells and also priming CD4^+^ T-cells (2, 6, 7). Liu et al. have developed a fast DNA intramuscular vaccination strategy that shortens the vaccination time to 5 days, but is able to elicit a stronger anti-tumor response independent of antigen doses in a murine lung cancer model compared to the traditional 2-week-interval immunization approach. Mechanistically, this new method–vaccination three times in a week, induces the accumulation of CD8^+^CD11c^+^ APCs, including both DCs and macrophages, in the vaccination site due to the local increased chemokines, thereby promoting antigen presentation and activation of CD8^+^ T-cells.

The utility of DCs for cancer therapy has been exemplified by the case report presented by Huang et al. A triple-regimen therapy entailing the intratumoral injection of low-dose autologous DCs and anti-PD-L1 antibody plus radiotherapy is given to a psoriatic patient with cutaneous squamous cell carcinoma (cSCC). A majority of these DCs are CD16^+^ myeloid DCs, an inflammatory DC subset, which are enriched through leukapheresis without *in vitro* and *in vivo* modification or expansion. The outcome with reduced tumor mass is promising, despite expected mild cutaneous side effects. Importantly, T-cell-related signatures are enriched in the biopsies, while activated anti-tumor innate and adaptive immune responses in the peripheral blood are observed post-therapy.

In addition to DCs and macrophages, B-cells can also function as APCs to stimulate T-cell response. However, the immunosuppressive tumor microenvironment often induces the differentiation of B-cells into regulatory B-cells (Bregs) that suppress T-cell activation and promote tumor growth. Interestingly, Li et al. have reported that triple negative breast cancer patients have the highest levels of a soluble form of programmed death ligand-1 (sPD-L1) and IL-10 in the serum along with the greater proportion of PD-1^+^ Bregs in the peripheral blood than patients with the other types of breast cancer. These results suggest that checkpoint blockade therapies targeting PD-1 and PD-L1 should take these suppressor B-cells into account.

Nanoparticles, such as liposomes, are good vaccine vectors, as they are able to deliver tumor antigens and adjuvant to activate DCs (8). Earlier results by Nijen Twilhaar et al. have shown that liposomes containing the ganglioside GM3, an endogenous ligand for CD169, specifically target CD169^+^ macrophages, and that co-injection of a soluble adjuvant induces strong cDC1-dependent CD8^+^ T-cell responses, largely *via* the antigen transfer from macrophages to cDC1 (9). Instead of supplementing with the soluble adjuvant that potentially causes adverse effects, the authors incorporated the toll-like receptor (TLR) ligands, TLR4 or TLR7/8, and/or inflammasome agonists into the GM3 liposomes by taking advantage of their adjuvanticity and potency to induce DC maturation and activation (10). The newly-developed liposomes remain to be efficiently taken up by CD169^+^ APCs, promote cDC1/cDC2 maturation and strongly activate CD4^+^ and CD8^+^ T-cells when used for immunization of mice along with a synthetic peptide. Interestingly, such effects are only induced by incorporated TLR ligands, but not inflammasome agonists, and no substantial additive effects are observed when both TLR ligands and inflammasome stimuli are incorporated. These results suggest that liposomal component and adjuvants need to be carefully selected when developing liposomes for cancer therapies.

In another study by Zhang et al., a DC/tumor cell fusion cell membrane (FCM) nano-vaccine (FCM-NP) is developed to treat ovarian cancer in preclinical murine models. FCM-NPs carry both the immunogenicity of tumor cells and the antigen-presenting capacity of DCs that is enhanced by the fusion process. The potent anti-cancer efficacy of such FCM-NPs has been proved by the delayed tumor growth and diminished metastasis associated with an expansion of tumor-specific cytotoxic CD8^+^ T-cells and reduced regulatory T-cells. Moreover, no substantial adverse effects are induced by this new nano-vaccine.

Lastly, Filin et al. have summarized new approaches and recent advances of DC-based cancer vaccines in clinical trials of various types of cancer. This review has also presented the methods of obtaining and activating DCs *in vitro* and *in vivo* for the generation of DC-based vaccines. A combination of DC vaccines with other immunotherapeutic approaches would be effective for cancer treatment, as suggested by the authors.

## Conclusions

This Research Topic “*Dendritic Cell-primed T Cells in Anti-tumor Immune Responses and Relevant Vaccine Strategies*” has stimulated the presentation and discussion of current research on the APC/DC-mediated regulation of tumor immunity and new APC/DC-based strategies for cancer treatment. Although this collection does not represent a complete list of current studies in this field, we (the editors) believe that each article published under this Research Topic will facilitate the future development of new or the improvement of existing APC/DC-based cancer immunotherapies.

## Author contributions

JWL wrote this editorial article. All authors made substantial, direct and intellectual contributions to the work, and approved it for publication.

## Funding

JWL is supported by the University of Alabama at Birmingham (UAB) faculty start-up funds, DoD W81XWH-18-1-0315 and NIH R01AI148711. CQ is supported by the Project of Jiangsu S&T Plan (BE2019651) and the Project of Changzhou S&T Plan (CE20225050).

## Acknowledgments

We would like to thank all authors for their contributions to this Research Topic.

## Conflict of interest

The authors declare that the research was conducted in the absence of any commercial or financial relationships that could be construed as a potential conflict of interest.

## Publisher’s note

All claims expressed in this article are solely those of the authors and do not necessarily represent those of their affiliated organizations, or those of the publisher, the editors and the reviewers. Any product that may be evaluated in this article, or claim that may be made by its manufacturer, is not guaranteed or endorsed by the publisher.
